# P-2067. Geographic Access to Liver Transplant Centers with HIV and HOPE Experience, 2017–2022

**DOI:** 10.1093/ofid/ofaf695.2231

**Published:** 2026-01-11

**Authors:** Hannah Rosenthal, Brett Fortune, Clara Tow, Hillary Yaffe, Vagish Hemmige

**Affiliations:** Albert Einstein College of Medicine, Bronx, NY; Montefiore Medical Center, Bronx, New York; Montefiore Medical Center, Bronx, New York; Montefiore, Bronx, New York; Montefiore Medical Center, Bronx, New York

## Abstract

**Background:**

People with HIV (PWH) face an increased risk of end-stage liver disease but continue to experience reduced access to liver transplantation. We aimed to assess whether geographic disparities persist in access to transplant centers experienced in transplanting PWH, with particular attention to centers participating in HIV Organ Policy Equity (HOPE) Act transplants.

**Figure:**
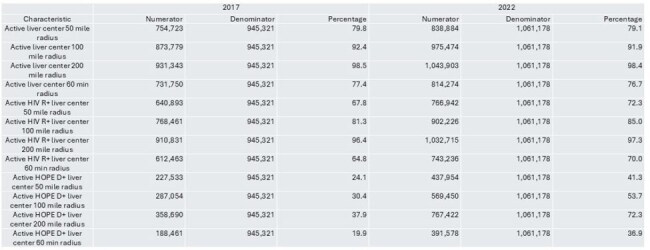
Access of PLWH to transplant centers in 2017 and 2022

**Figure:**
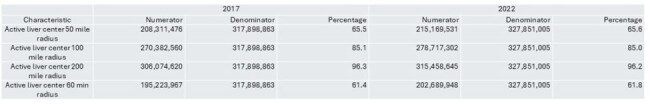
Access of people without HIV to transplant centers in 2017 and 2022

**Methods:**

We used CDC county-level HIV prevalence data from 2017 and 2022 and extrapolated these to the census tract level using population-weighted estimates. Transplant center locations were identified using HRSA data, and liver transplant activity was derived from SRTR. Centers were categorized by whether they performed liver transplants in PWH or used HIV-positive donors (HOPE donors) during 2013–2017 and 2018–2022. We calculated physical (50-, 100-, 200-mile) and driving-time (60-minute) radii from each center to estimate the number of PWH and non-HIV individuals within geographic access zones.

**Figure:**
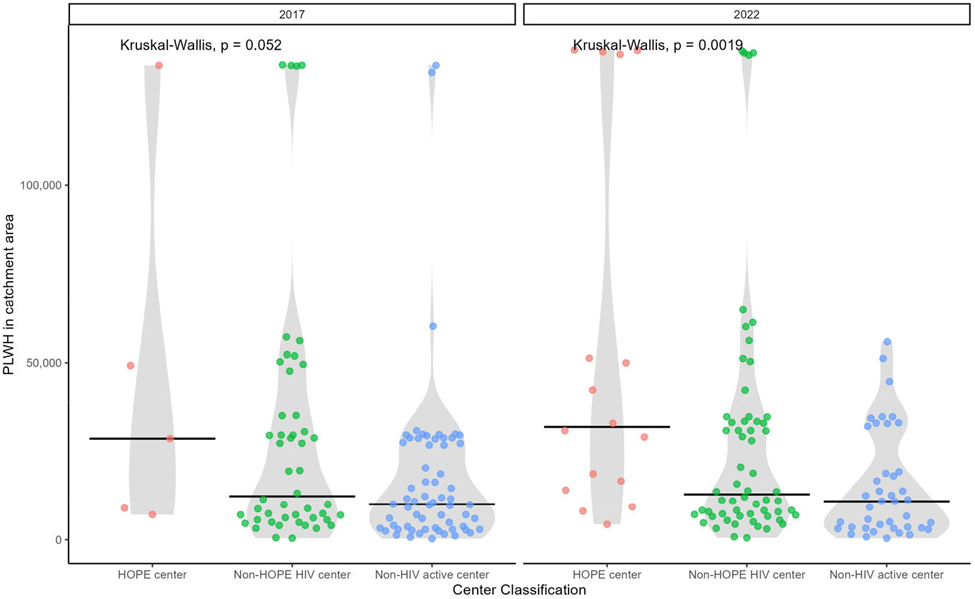
PWH within 50 mile radius of liver transplant centers, 2017 and 2022

**Figure:**
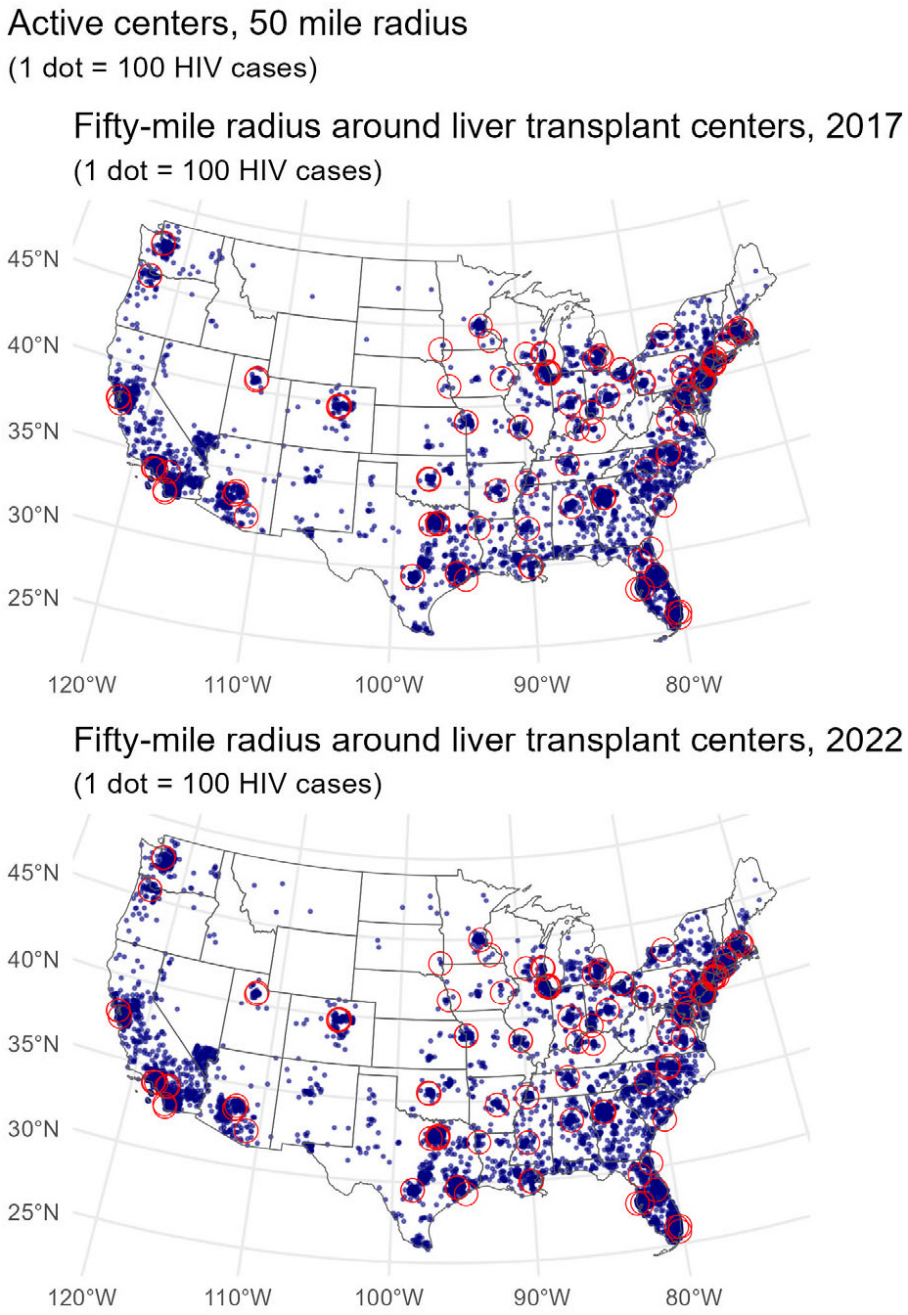
PWH in the United States and 50-mile radii of active liver transplant centers, 2017 and 2022

**Results:**

In both 2017 and 2022, approximately 80% of PWH lived within 50 miles of any liver transplant center. Access to centers with experience in transplanting PWH improved from 67.8% to 72.3%, surpassing access for people without HIV. Access to HOPE-experienced centers also improved significantly over time and distance. Kruskal-Wallis testing showed significant differences in the distribution of PWH within the 50-mile radius of HIV D+ experienced centers, HIV D– experienced centers, and centers with neither experience—primarily driven by greater proximity to HOPE centers.

**Conclusion:**

While geographic access to transplant centers with HIV and HOPE experience has improved, particularly from 2017 to 2022, physical proximity alone is unlikely to be the main barrier to transplant in PWH. Further research is needed to identify and address systemic and structural barriers that limit transplant access for this vulnerable population.

**Disclosures:**

Brett Fortune, MD, Cook Medical: Honoraria|W.L. Gore & Associates: Honoraria Vagish Hemmige, MD, Merck: Grant/Research Support

